# Teacher resilience and adaptation in digital teaching contexts: a systematic review of research trends, psychological constructs, and evidence gaps

**DOI:** 10.3389/fpsyg.2026.1950412

**Published:** 2026-09-15

**Authors:** Yao Yao

**Affiliations:** Tongling Polytechnic, Tongling, Anhui, China

**Keywords:** digital competence, digital teaching, emergency remote teaching, MMAT, systematic review, teacher adaptation, teacher resilience

## Abstract

Digital teaching expanded rapidly during the COVID-19 emergency and continues through hybrid, robot-mediated, metaverse, and artificial-intelligence-supported education. Yet evidence about teacher resilience and adaptation remains conceptually and methodologically dispersed. This PRISMA 2020-guided systematic review mapped how resilience and adaptation are conceptualized, which psychological constructs and indirect statistical pathways have been studied, and where important evidence gaps remain. Web of Science and Scopus records exported on 21 and 23 February 2026 yielded 4,465 records; 27 English-language empirical studies were included after duplicate removal, screening, retrieval, and full-text eligibility assessment. Data were extracted into a structured matrix, synthesized descriptively and thematically, and appraised with the Mixed Methods Appraisal Tool (MMAT) 2018. The corpus comprised 11 qualitative, 9 quantitative non-randomized, 5 mixed-methods, and 2 quantitative descriptive studies, involving at least 11,199 teachers or teacher educators. Twenty-one studies concerned COVID-19/emergency remote teaching and six examined emerging technologies. Six non-mutually-exclusive themes were identified: personal psychological resources and coping (22/27), pedagogical adaptation, agency, and innovation (21/27), digital competence, self-efficacy, and readiness (19/27), contextual and structural constraints (18/27), wellbeing-related outcomes (16/27), and social or institutional support (13/27). Seven studies tested indirect effects, but all used cross-sectional quantitative components; none formally tested contextual moderation or evaluated an intervention. MMAT findings supported qualitative coherence while highlighting confounding, representativeness, nonresponse, and weakest-component limitations in quantitative and mixed-methods evidence. The evidence therefore supports a context-embedded descriptive account of resilience and adaptation. Future research should prioritize longitudinal, intervention, and explicitly technology-specific designs.

**Systematic review registration:**

https://osf.io/xf2ve/overview, xf2ve.

## Introduction

Digital teaching is no longer limited to emergency remote instruction. Schools and universities now work across online, hybrid, learning-management, robot-mediated, metaverse, and artificial-intelligence-supported environments. These environments can extend access and pedagogical options, but they also redistribute teachers’ workload, require rapid technical and pedagogical learning, alter professional roles, and intensify uncertainty. Teacher resilience and adaptation have consequently become prominent constructs for understanding how educators sustain practice and wellbeing during technological change (Kidd and Murray, 2020; Alhawsawi et al., 2023; Ahumada-Newhart and Eccles, 2025; Liu and Chang, 2024).

The two concepts overlap but are not interchangeable. Resilience can be framed as a relatively stable personal resource, a dynamic process of mobilizing resources under adversity, or an outcome expressed in sustained functioning (Mansfield et al., 2012). Adaptation more often denotes behavioral, pedagogical, cognitive, or emotional adjustment to changed teaching conditions (Shi et al., 2025). Distinguishing these positions is analytically important because studies may locate resilience or adaptation as a resource, a process, an intervening construct, or an outcome, leading to different forms of evidence and interpretation.

Existing reviews establish important but fragmented knowledge. Crompton et al. (2022) mapped support provided to K–12 teachers during emergency remote education; Sum and Oancea (2022) synthesized academics’ technology use during higher-education emergency remote teaching; and Kaqinari (2023) reviewed facilitators and barriers reported by university lecturers. Collectively, these reviews show that technological adjustment depends on both teacher capabilities and institutional conditions. Their review objects, however, are support, technology use, or barriers within particular sectors and emergency contexts—not the conceptual architecture of teacher resilience and adaptation across education levels. Consequently, they do not show how resilience and adaptation are defined and operationalized across mixed study designs, nor do they distinguish frequently discussed psychological or contextual factors from constructs tested in formal indirect-effect or moderation models. These unresolved questions directly motivate RQ2 and RQ3, while cross-sector coverage and design-specific quality appraisal motivate RQ1 and RQ4.

Accordingly, this mixed-studies systematic review compares how resilience and adaptation are defined and operationalized, synthesizes the psychological constructs, contextual conditions, teacher outcomes, and formally tested indirect statistical pathways reported across studies, and appraises methodological quality using MMAT. The synthesis distinguishes descriptive thematic coverage from formally tested statistical relationships and considers evidence from both pandemic-era emergency teaching and emerging digital-technology contexts.

### Review questions

1. *RQ1*: What are the publication, educational-context, and methodological characteristics of empirical research on teacher resilience and adaptation in digital teaching contexts?
2. *RQ 2*: How are resilience and adaptation conceptualized and operationalized in the included studies?
3. *RQ 3*: Which psychological constructs, formally tested indirect statistical pathways, contextual conditions, and teacher outcomes are reported?
4. *RQ 4*: Which theoretical, methodological, and contextual gaps should guide future research?

## Methods

### Review design, reporting, and protocol status

The review followed the PRISMA 2020 reporting statement (Page et al., 2021). It is described as a mixed-studies systematic review because it synthesizes findings across qualitative, quantitative, and mixed-methods studies and formally appraises methodological quality; descriptive mapping is one component rather than the sole objective. No review protocol was prospectively registered. This absence is reported transparently and considered in the limitations because analytic decisions could not be compared with a time-stamped protocol.

### Eligibility framework

An adapted PICOS logic was used because the corpus included qualitative, quantitative, and mixed-methods designs and did not require an intervention or comparator. A report had to contain original empirical evidence from teachers or teacher educators and directly examine resilience, adaptation, or psychological adjustment in a digital-teaching context. Outcomes were not prespecified as eligibility criteria; instead, associated coping, competence, agency, wellbeing, and work outcomes were coded inductively after inclusion.

### Information sources and search strategy

The bibliographic search used Web of Science Core Collection and Scopus. The Web of Science search was run in the Topic field on 21 February 2026 and yielded 3,174 records. The Scopus search was run in All fields on 23 February 2026 and yielded 1,291 records. Both databases used equivalent concept blocks for the teacher population, resilience or adaptation, and digital-teaching context.

The common Boolean logic was: (teacher resilience* OR educator resilience* OR teacher adapt OR educator adapt) AND (online OR remote OR digital OR technology assisted teaching OR AI). Platform-specific field tags and syntax were adapted to Web of Science and Scopus.

The retained search notes preserve the common Boolean logic, database fields, search dates, and database-specific yields, although the complete platform-generated search histories were not retained. Supplementary File S1 records the search audit and treats the two archived database exports as the sole source of the PRISMA identification total. No additional information source contributed records to the final review.

### Record management and study selection

The two database exports yielded 4,465 records in total. After 1,883 duplicate records were removed, 2,582 unique records underwent title-and-abstract screening. Screening excluded 2,457 records, leaving 125 reports sought for retrieval. One report could not be retrieved; therefore, 124 reports were retrieved and assessed for eligibility at the full-text stage. Ninety-seven full texts were excluded using one mutually exclusive primary reason: not teacher practice (*n* = 5), no direct teacher resilience/adaptation focus (*n* = 58), no relevant teaching-technology context (*n* = 14), teacher not the unit or subject of analysis (*n* = 13), non-empirical study (*n* = 1), resilience at a non-teacher level (*n* = 3), no direct resilience/adaptation construct (*n* = 2), or full text not in English (*n* = 1). Twenty-seven empirical studies remained.

Title-and-abstract screening and full-text eligibility decisions were led by the primary author. A professor volunteered to assist with data extraction and data processing but did not serve as an independent second screener. Eligibility decisions were subsequently re-audited manually against the final criteria, and uncertain cases were resolved by re-examining the eligibility rule and the source report. Because screening was not conducted independently in duplicate, no screening-agreement statistic was calculated; the possible risk of selection bias is acknowledged below.

### Data extraction and coding

Data extraction and processing were conducted by the primary author with assistance from a professor who volunteered to support the review. The professor helped organize study information, verify source evidence, process the dataset, and review uncertain extraction or coding entries through discussion and re-examination of the full-text reports. This assistance strengthened data checking but did not constitute an independently duplicated extraction; therefore, no inter-extractor reliability statistic was calculated.

A structured matrix recorded study ID, citation, year, country, setting, education group, primary teacher sample, design, data collection, analysis, digital context, construct route, conceptualization, findings, theme codes, quality judgments, and source locator. Primary sample size refers to the principal teacher or teacher-educator analytic sample; the summed total is descriptive and is not treated as a pooled independent sample. One study with 610 questionnaire starts was coded as 525 valid analytic cases after stated quality exclusions.

Coding was iterative and audit-oriented. First, line-level descriptive codes were assigned to constructs, teacher responses, contextual conditions, and outcomes. Second, related codes were grouped into descriptive themes. Third, analytical distinctions were added between resilience conceptualization, broad thematic association, formal indirect-effect testing, and contextual conditions. Theme boundaries and study assignments were checked against the full text and then reconciled with the extraction matrix. Coding was completed in spreadsheets rather than dedicated qualitative-analysis software. Frequencies represent the number of studies contributing evidence to a theme and are non-mutually exclusive (see Figure 1).

**Figure 1:**
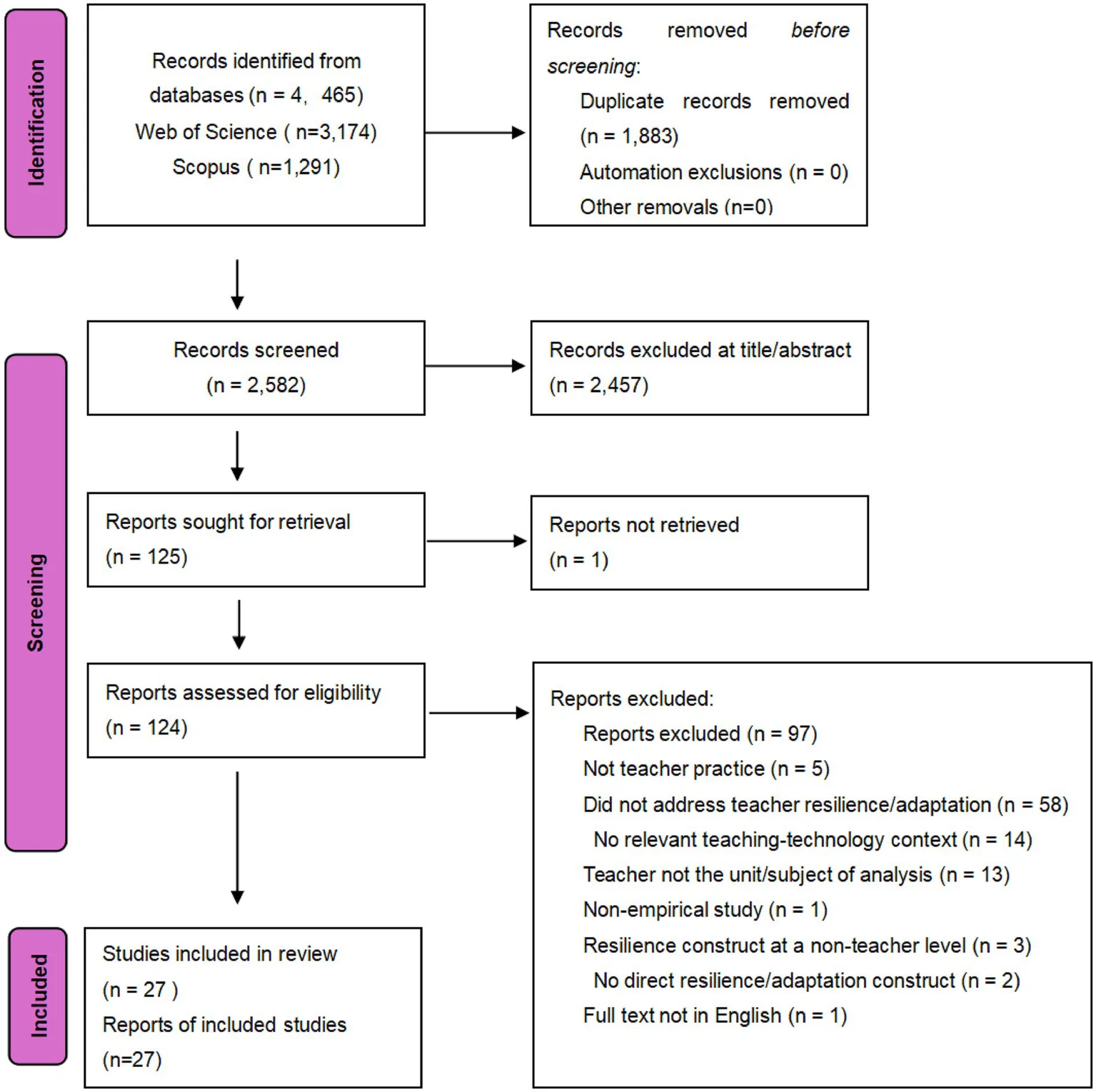
PRISMA 2020 flow diagram for the final 27-study corpus. Adapted from the PRISMA 2020 flow diagram template (Page et al., 2021), distributed under CC BY 4.0.

### Methodological quality appraisal

The Mixed Methods Appraisal Tool, version 2018 (MMAT), was applied because it provides design-specific criteria for qualitative, quantitative non-randomized, quantitative descriptive, and mixed-methods research (Hong et al., 2018). Each study received two screening judgments (S1–S2) and five design-specific judgments (C1–C5), rated Yes (Y), No (N), or Cannot tell (CT). Mixed-methods criterion 5.5 followed the weakest-component principle. No numerical total quality score was calculated, and no study was excluded solely because of MMAT findings. The primary author completed a second full-text check of all N/CT judgments. As part of the professor volunteer’s assistance with data verification, the supporting source evidence and explanations for these judgments were also reviewed collaboratively. Uncertain decisions were resolved through discussion and re-examination of the full text against the applicable MMAT criterion. This was not a fully independent duplicate appraisal, so no inter-rater reliability statistic was calculated. The complete study-level matrix is provided in Supplementary File S1.

### Synthesis

Descriptive counts and percentages summarized year, design, context, education group, construct route, themes, indirect-effect studies, and MMAT judgments. Thematic synthesis followed the logic of coding text, developing descriptive themes, and generating higher-order analytical interpretations (Thomas and Harden, 2008). Because designs, constructs, measures, and outcomes were heterogeneous, no meta-analysis was attempted. A finding was classified as a formal indirect pathway only when the primary study reported an explicit mediation, indirect-effect, or serial-path test. Contextual variables were classified as moderators only when a formal interaction or conditional-effect test was reported. This rule prevented descriptive support or institutional conditions from being mislabeled as statistical moderation (see Table 1).

**Table 1:** Adapted PICOS framework and eligibility criteria.

Element	Operational definition	Inclusion	Exclusion
Population	Practicing teachers, teacher educators, lecturers/faculty, or instructors directly engaged in teaching.	Teacher/teacher-educator data are a primary analytic unit.	Students, administrators, organizations, or communities without separable teacher-level evidence; pre-service students not simultaneously teaching.
Interest/exposure	Digital, online, remote, hybrid, ICT-, robot-, metaverse-, or AI-mediated teaching.	The technological context materially shapes teaching practice or adjustment.	Technology used only for data collection or student learning without a teacher-practice focus.
Comparator	Not required.	Any comparator or no comparator.	None on comparator grounds.
Construct/outcomes	Direct resilience, adaptation, or psychological-adjustment construct; associated coping, competence, agency, wellbeing, or work outcomes coded after inclusion.	A direct construct is measured or substantively analyzed.	Only tangential mentions; resilience at a student, organizational, or community level; no direct resilience/adaptation construct.
Study design	Original empirical qualitative, quantitative, or mixed-methods research available in English.	Sufficient full-text methods and results for extraction and appraisal.	Reviews, conceptual papers, commentaries, protocols, non-empirical reports, or non-English full text.
Publication period	2020–2026.	Record falls within the accelerated digital-transformation period represented in the archived search exports.	Published outside the specified period.

## Results

### Study characteristics and methodological quality (RQ1)

The 27 studies were published from 2020 to 2026: one in 2020, two in 2021, three in 2022, six in 2023, four in 2024, nine in 2025, and two in 2026. The methods comprised 11 qualitative studies (40.7%), nine quantitative non-randomized studies (33.3%), five mixed-methods studies (18.5%), and two quantitative descriptive studies (7.4%). Twenty-one studies (77.8%) examined COVID-19 or emergency remote teaching, whereas six (22.2%) examined emerging technologies. Fourteen studies involved higher, teacher, or technical education, 12 involved school or early-childhood education, and one involved cross-sector online language teaching. Across reported primary teacher samples, the descriptive lower-bound total was 11,199.

Twelve studies used resilience as the primary construct, nine focused on adaptation, and six addressed both. Table 2 presents every included study, including its setting, primary sample, design, construct route, and main contribution.

**Table 2:** Characteristics of all 27 included studies.

ID	Author-year	Country/setting	Primary n	Design/MMAT category	Construct	Digital context	Main contribution
S01	Abdul-Majied and Figaro-Henry (2024)	Trinidad and Tobago—Early childhood	6	Qualitative descriptive case study	Both	COVID/ERT	Teachers sustained instruction by combining Zoom, Padlet, collaboration and iterative problem solving while managing access and engagement barriers.
S02	Ahumada-Newhart and Eccles (2025)	United States—K-12	60	National qualitative multi-case study	Adaptation	Emerging technology	Robot-mediated learning reconfigured teachers as facilitators, coordinators and social/technical mediators, with persistent inclusion and collaboration challenges.
S03	Alhawsawi et al. (2023)	Saudi Arabia—Higher education	5	Qualitative case analysis reported from a wider mixed-method project	Resilience	COVID/ERT	Personal and contextual resources shaped coping and resilience during the rapid online transition, with institutional and gendered constraints.
S04	Bavli and Kortel (2023)	Turkey—K-12 and mixed school levels	15	Qualitative phenomenological study	Resilience	COVID/ERT	Teacher resilience appeared across professional, social, emotional and motivational dimensions and was strengthened by support, communication and purposeful persistence.
S05	Cahapay and Bangoc (2022)	Philippines—K-12	2,272	Cross-sectional analytical survey with mediation model	Resilience	COVID/ERT	Stress was negatively associated with life satisfaction, while hope and resilience statistically mediated the association.
S06	Craw and Bevan (2022)	United States—K-12	163	Sequential mixed-method study	Resilience	COVID/ERT	Ambiguous loss was linked to stress, while communal coping, reassurance and validation supported resilience.
S07	Williams et al. (2023)	South Africa—Urban K-12 schools	240	Sequential explanatory mixed-method study	Resilience	COVID/ERT	Problem-focused coping, planning and related cognitive strategies were positively associated with teacher resilience and explained through qualitative accounts.
S08	Eşki et al. (2025)	Spain—Teacher education/higher education	3	Longitudinal qualitative multiple-case study	Both	COVID/ERT	Digital literacy developed beyond technical skill toward pedagogical, critical and ethical competence, supported by reflection and professional learning.
S09	Gao et al. (2025)	China—Higher education	373	Cross-sectional analytical survey with SEM mediation	Adaptation	Emerging technology	Psychological adaptability predicted work engagement both directly and indirectly through digital-information-literacy self-efficacy.
S10	Colombo-Gomes (2026)	Brazil—Pre-service teacher education plus active private-school teaching	5	Qualitative case study	Both	COVID/ERT	Participants used emotional labour and meaning-making to sustain teaching and build resilience amid persistent post-crisis structural pressures.
S11	Hujala et al. (2025)	Finland—Higher education	358	Convergent survey-based mixed-method study	Adaptation	COVID/ERT	Teaching beliefs and intentions interacted with institutional conditions to shape emergency-remote-teaching actions and adaptation.
S12	Khojasteh et al. (2025)	Iran—Medical higher education	130	Cross-sectional analytical survey with supplementary open-ended responses	Both	COVID/ERT	Medical educators showed moderate change attitudes and resilience while reporting technological, organizational and pedagogical barriers.
S13	Kidd and Murray (2020)	England, United Kingdom—Teacher education	14	Rapid-response qualitative study	Adaptation	COVID/ERT	Teacher educators moved practicum learning into online spaces through pedagogic agility, technology-enabled care and redesigned professional learning.
S14	Kowitarttawatee and Limphaibool (2022)	Thailand—Higher education	330	Concurrent triangulation mixed-method study	Resilience	COVID/ERT	Western mindfulness dimensions were positively related to teacher resilience, while qualitative accounts emphasized mindful coping and institutional practice.
S15	Laidlaw (2023)	Canada—School music education	218	Concurrent mixed-method study	Resilience	COVID/ERT	Music teachers reported severe workload and wellbeing pressures alongside adaptive practice, peer support and resilience resources.
S16	Lizárraga Sánchez et al. (2025)	Mexico—Higher education	74	Descriptive quantitative case survey	Both	COVID/ERT	Teachers perceived ICT use and resilience as supporting digital adaptation, while infrastructure, training and workload remained barriers.
S17	Makrakis (2024)	Indonesia, Malaysia and Vietnam—Primary and secondary education	1,815	Cross-sectional scale-development and validation survey	Resilience	Emerging technology	A two-factor ICT/metaverse resilience structure was supported, and self-efficacy and transformative teaching beliefs predicted resilience scores.
S18	Mehta (2021)	India—Senior secondary private schools	388	Cross-sectional analytical survey using PLS-SEM	Adaptation	COVID/ERT	Readiness for online teaching was shaped by technology-acceptance beliefs and was related to perceived stress during lockdown.
S19	Padmanabhanunni et al. (2023)	South Africa—K-12 and mixed school levels	355	Cross-sectional analytical survey with structural model	Resilience	COVID/ERT	Resilience was associated with better life satisfaction and fewer adverse psychological outcomes and statistically mediated role-stress relationships.
S20	Papazis et al. (2023)	Greece—Primary education	1,415	Cross-sectional analytical survey	Resilience	COVID/ERT	Higher resilience was associated with more adaptive attitudes toward emergency remote teaching and lower perceived stress.
S21	Rodríguez-Hidalgo et al. (2024)	Spain—K-12	289	Cross-sectional analytical survey with SEM	Resilience	COVID/ERT	Resilience and engagement functioned as positive predictors in a model of teacher self-efficacy, whereas burnout dimensions were disruptive.
S22	Saikrishna (2025)	India—Higher education	14	Qualitative interpretive phenomenological study using Gioia methodology	Adaptation	Emerging technology	Educator roles shifted toward facilitation, mentoring, data-informed customization and culturally inclusive adaptive learning.
S23	Shi et al. (2025)	China—Online language teaching	525	Cross-sectional analytical survey with SEM	Resilience	Emerging technology	Psychological resilience and digital competence were positively associated with online-teaching self-efficacy.
S24	Wickersham et al. (2026)	United States—Technical college	14	Qualitative case study	Both	COVID/ERT	Pandemic disruption accelerated instructional innovation and pedagogical resilience through determination, endurance, collaboration and redesigned technical learning.
S25	Xu and Tao (2025)	China—Higher education	4	Longitudinal qualitative multiple-case study	Adaptation	COVID/ERT	Pedagogical and socio-affective identity tensions triggered different forms of teacher agency and adaptive online-teaching practice.
S26	Liu and Chang (2024)	China—Higher education	10	Longitudinal qualitative study	Adaptation	Emerging technology	Positive emotions supported adaptive expertise as teachers negotiated AI-related pedagogical change, uncertainty and professional growth.
S27	Zou et al. (2021)	China—Higher education	2,104	Cross-sectional analytical survey using latent profile analysis	Adaptation	COVID/ERT	Distinct affect profiles were associated with different levels of psychological adjustment to online teaching.

All 27 studies met both MMAT screening questions. Across the 135 design-specific criteria, 110 judgments were Y (81.5%), 20 were N (14.8%), and five were CT (3.7%). Eleven studies met all five design-specific criteria; 16 had at least one N or CT. All 11 qualitative studies met all five qualitative criteria. Each of the nine quantitative non-randomized studies received N for confounder adjustment (C4), reflecting principal cross-sectional models that did not adequately address plausible confounding. Both quantitative descriptive studies received N for representativeness (C2) and CT for nonresponse bias (C4). All five mixed-methods studies had N or CT on criterion 5.5 under the weakest-component rule (four N, one CT).

The appraisal does not imply that the 16 studies with N/CT are unusable. It calibrates the findings: qualitative evidence was generally coherent for understanding experience and context, whereas quantitative associations and indirect effects require caution about sampling, nonresponse, confounding, and temporal ordering. No study was excluded based on MMAT results (see Table 3).

**Table 3:** MMAT Round-2 summary by design category.

MMAT category	Studies	All five criteria Y	At least one N/CT
Qualitative	11	11	0
Quantitative non-randomized	9	0	9
Quantitative descriptive	2	0	2
Mixed methods	5	0	5
Total	27	11	16

Criteria-only rating totals: *Y* = 110 (81.5%), *N* = 20 (14.8%), and CT = 5 (3.7%). No summative overall MMAT score was calculated. Full study-level judgments and decision notes are available in Supplementary File S1.

### Conceptualization and operationalization (RQ2)

Among the 18 resilience or combined-construct studies, nine (50.0%) primarily treated resilience as a dynamic or context-embedded process, seven (38.9%) treated it as an individual psychological resource or capacity, and two (11.1%) used an outcome- or technology−/relationship-specific construct. The nine adaptation-only studies were not forced into a resilience typology. Qualitative studies commonly represented resilience through coping, persistence, reflection, collaboration, and pedagogical reconfiguration (Abdul-Majied and Figaro-Henry, 2024; Kidd and Murray, 2020; Williams et al., 2023), whereas quantitative studies more often operationalized it through standardized psychological scales, structural models, or technology-specific measurement development (Cahapay and Bangoc, 2022; Makrakis, 2024; Papazis et al., 2023; Shi et al., 2025).

Operationalizations also differed in direction. Resilience appeared as an antecedent of life satisfaction, self-efficacy, or lower distress, as an intervening construct, and as an outcome associated with mindfulness or technological beliefs (Kowitarttawatee and Limphaibool, 2022; Padmanabhanunni et al., 2023; Rodríguez-Hidalgo et al., 2024). Adaptation ranged from readiness and psychological adjustment to observed changes in pedagogy, professional identity, or educator roles (Liu and Chang, 2024; Mehta, 2021; Xu and Tao, 2025; Zou et al., 2021). These differences support reporting construct routes explicitly rather than aggregating them as equivalent measures.

### Psychological patterns, contextual conditions, and tested pathways (RQ3)

Six non-mutually-exclusive themes summarized the 27 studies (Figure 2 and Table 4). Personal psychological resources and coping appeared in 22 studies (81.5%), followed by pedagogical adaptation, agency, and innovation in 21 (77.8%), digital competence, self-efficacy, and readiness in 19 (70.4%), contextual and structural constraints in 18 (66.7%), wellbeing, stress, burnout, and engagement outcomes in 16 (59.3%), and social, collegial, and institutional support in 13 (48.1%). The personal-resource theme included hope, mindfulness, affect, and coping (Cahapay and Bangoc, 2022; Kowitarttawatee and Limphaibool, 2022; Williams et al., 2023; Zou et al., 2021). Pedagogical adaptation and digital capability were represented through redesigned practicum, identity work, adaptive expertise, readiness, and technology-specific confidence (Eşki et al., 2025; Kidd and Murray, 2020; Liu and Chang, 2024; Makrakis, 2024; Mehta, 2021; Shi et al., 2025; Xu and Tao, 2025). These frequencies indicate coverage only; they do not establish effect magnitude, consistency of direction, or certainty.

**Figure 2:**
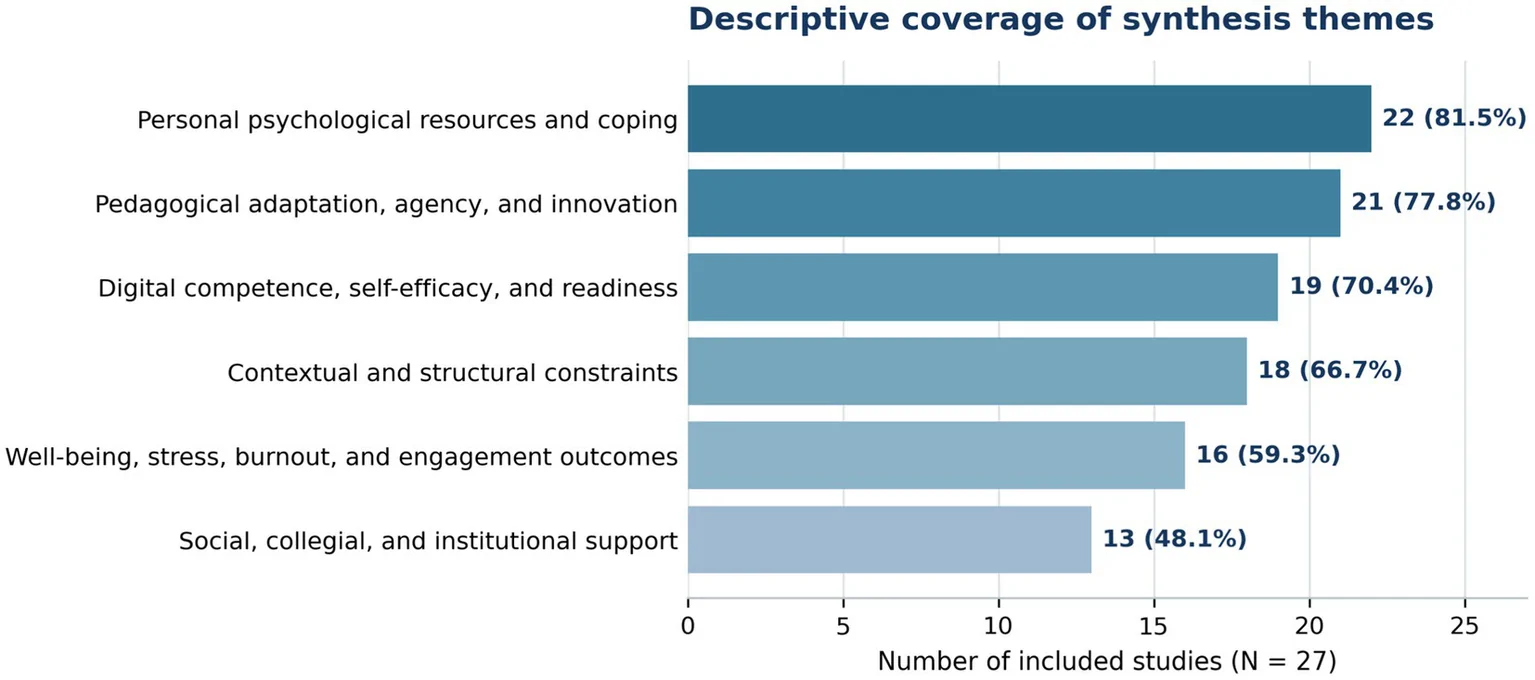
Descriptive coverage of non-mutually-exclusive synthesis themes. Themes were not manually exclusive. Frequencies indicate study coverage, not effect size or certainty.

**Table 4:** Thematic synthesis and study coverage.

Code	Theme	*n* (%)	Included study IDs	Interpretive summary
T1	Personal psychological resources and coping	22 (81.5%)	S01, S03, S04, S05, S06, S07, S08, S09, S10, S12, S13, S14, S15, S16, S17, S19, S20, S21, S23, S24, S26, S27	Hope, mindfulness, affect, meaning-making, coping, persistence, and related personal resources were examined as supports or correlates of resilience/adaptation.
T2	Digital competence, self-efficacy, and readiness	19 (70.4%)	S01, S02, S03, S04, S08, S09, S11, S12, S13, S15, S16, S17, S18, S20, S22, S23, S24, S26, S27	Digital competence, self-efficacy, technology beliefs, and readiness shaped teachers’ reported confidence and adjustment.
T3	Pedagogical adaptation, agency, and innovation	21 (77.8%)	S01, S02, S03, S04, S07, S08, S09, S10, S11, S12, S13, S16, S17, S18, S20, S22, S23, S24, S25, S26, S27	Teachers redesigned pedagogy, roles, routines, identities, or technological practices through experimentation and agency.
T4	Social, collegial, and institutional support	13 (48.1%)	S01, S02, S03, S04, S06, S07, S08, S13, S14, S15, S22, S24, S25	Peer, student, leadership, and institutional support supplied relational or organizational resources; these were contextual conditions, not formal moderators.
T5	Wellbeing, stress, burnout, and engagement outcomes	16 (59.3%)	S04, S05, S06, S07, S09, S10, S14, S15, S18, S19, S20, S21, S23, S25, S26, S27	Studies related resilience/adaptation to stress, burnout, life satisfaction, engagement, psychological adjustment, or self-efficacy outcomes.
T6	Contextual and structural constraints	18 (66.7%)	S01, S02, S03, S04, S06, S07, S08, S10, S11, S12, S13, S15, S16, S18, S19, S22, S24, S25	Access, workload, time, policy, infrastructure, role ambiguity, and contextual inequities constrained available adaptive responses.

Contextual constraints and support were reported together rather than as competing explanations. Workload, access, infrastructure, time, policy, role ambiguity, and unequal responsibilities limited available responses, while collegial, student, leadership, and institutional support provided relational or organizational resources (Ahumada-Newhart and Eccles, 2025; Craw and Bevan, 2022; Hujala et al., 2025; Laidlaw, 2023). Wellbeing-related findings included stress, burnout, engagement, life satisfaction, psychological adjustment, and teacher self-efficacy (Mehta, 2021; Padmanabhanunni et al., 2023; Papazis et al., 2023; Rodríguez-Hidalgo et al., 2024).

Seven studies (25.9%) reported formal indirect-effect tests involving hope, resilience, communal coping, stress, digital-information-literacy self-efficacy, mindfulness, readiness to change, role ambiguity, or digital competence (Cahapay and Bangoc, 2022; Craw and Bevan, 2022; Gao et al., 2025; Kowitarttawatee and Limphaibool, 2022; Mehta, 2021; Padmanabhanunni et al., 2023; Shi et al., 2025). Every model used a cross-sectional quantitative component. Their temporal order was proposed rather than observed, and MMAT identified additional concerns about representativeness or confounder adjustment in several studies. The results are therefore described as indirect statistical pathways, not demonstrated causal mechanisms.

No included study formally tested contextual moderation. Social support, leadership, access, workload, time, gendered responsibilities, culture, and institutional policy appeared as contextual conditions in qualitative accounts, group comparisons, or descriptive associations. Relabeling these factors as “moderators” would exceed the statistical evidence. No intervention trial evaluated whether a resilience, digital-competence, mindfulness, or organizational-support program caused improvement in teacher outcomes.

### Evidence gaps (RQ4)

Four gaps followed directly from the corpus. First, temporal and causal evidence was absent: the seven indirect-effect studies were cross-sectional, no intervention was evaluated, and no contextual moderator was formally tested. Second, the context base remained historically concentrated: 21 of 27 studies concerned COVID-19 or emergency remote teaching, whereas only six addressed robot-mediated, metaverse, artificial-intelligence, or other emerging-technology settings (Ahumada-Newhart and Eccles, 2025; Gao et al., 2025; Liu and Chang, 2024; Makrakis, 2024; Saikrishna, 2025; Shi et al., 2025). Third, setting coverage was uneven: 14 studies involved higher, teacher, or technical education, compared with 12 school or early-childhood studies and one cross-sector study; evidence remained sparse for special education, rural, low-resource, and cross-cultural comparisons. Fourth, measurement and design were fragmented: resilience and adaptation were operationalized in different directions, while 16 studies had at least one design-specific MMAT N/CT judgment. These gaps delimit what the review can explain and form the basis of the research agenda below.

## Discussion

### Principal findings

This review presents a structured synthesis of a heterogeneous evidence base on teacher resilience and adaptation in digital teaching. The final corpus is dominated by emergency remote teaching, cross-sectional quantitative designs, and qualitative accounts of rapid adaptation. Across methods, teacher responses were most often described through personal resources and coping, pedagogical agency, digital competence, contextual constraints, wellbeing-related outcomes, and social or institutional support. The relative frequency of these themes supports a multi-level, context-embedded interpretation but does not determine causal order.

The distinction between thematic coverage and formal path testing changes the substantive conclusion. Seven studies reported indirect effects, yet all were cross-sectional and none established temporal mediation. Moreover, no study formally tested contextual moderation involving leadership, social support, time, gender, or culture. The evidence therefore supports a set of plausible, heterogeneous statistical pathways situated within recurring contextual conditions, but it does not establish a single general explanatory model.

### Conceptual contribution relative to prior reviews

Prior reviews show that technology use, support, competence, and barriers are interdependent during emergency teaching (Crompton et al., 2022; Sum and Oancea, 2022; Kaqinari, 2023). The present review contributes a narrower construct-level distinction. First, resilience is split between dynamic/contextual and individual-resource conceptualizations rather than treated as one trait. Second, adaptation is retained as a related but distinct route rather than automatically relabeled resilience. Third, descriptive resources and constraints are separated from formally tested indirect effects and moderators. Fourth, MMAT findings are integrated into the interpretation of psychological models rather than presented as an isolated checklist.

This distinction is particularly important as research moves beyond emergency remote teaching. Emerging-technology studies described robot-mediated teacher roles, metaverse-related resilience, artificial-intelligence-related adaptive expertise, digital competence, and online-teaching self-efficacy. These topics suggest that future resilience research should examine the durability of professional identity, agency, and pedagogical judgment under continuing technological change. The present corpus is too small and heterogeneous, however, to claim an AI-specific resilience theory.

### Implications for practice and policy

The synthesis supports a portfolio approach to teacher support. Individual learning opportunities should integrate technical capability, pedagogical redesign, confidence-building, coping, and reflective practice. Organizational action should address workload, planning time, infrastructure, technical assistance, role clarity, and access to collegial learning. These recommendations are justified as responses to recurring reported conditions; they are not claims that a specific program has been proven effective.

Institutions should also avoid treating resilience as a substitute for adequate working conditions. When adaptive performance is achieved through uncompensated time, personal devices, blurred work–life boundaries, or repeated improvisation, “resilience” may conceal structural burden. Evaluation should therefore include both teacher resources and the conditions that create or reduce avoidable demands.

### Research agenda

- Use prospective longitudinal designs with repeated measures to establish temporal order among demands, appraisals, resources, adaptation, and outcomes.
- Test clearly specified interventions using comparison conditions, implementation data, and teacher-centered outcomes rather than assuming that training improves resilience.
- Distinguish statistical mediation from causal mediation and adjust plausible confounding in structural and path models.
- For future hypothesis-testing studies, pre-register hypotheses, models, primary outcomes, and moderator tests, and report sampling frames, response denominators, missingness, and robustness checks.
- Develop construct-valid technology-specific measures only when they add explanatory value beyond general resilience, self-efficacy, or technology-acceptance measures.
- Rebalance the evidence base toward routine, post-emergency, and emerging-technology teaching contexts, while treating pandemic-era studies as an important historical foundation rather than the field’s continuing default; include early-childhood, technical/vocational, special-education, rural, low-resource, and cross-cultural settings.
- Study organizational conditions as testable exposures or moderators while preserving qualitative inquiry into how teachers interpret those conditions (see Table 5).

**Table 5:** Formally tested indirect statistical pathways.

Study	Antecedent	Intervening construct	Outcome	Reported result	Design-based limit
Cahapay and Bangoc (2022)	Perceived stress	Hope and resilience (parallel)	Satisfaction with life	Both reported indirect effects were statistically significant.	Temporal order was not observed; mediation is statistical, not causal.
Craw and Bevan (2022)	Ambiguous loss	Perceived stress and communal coping (serial)	Interpersonal resilience	The full serial indirect effect was statistically significant.	Cross-sectional path analysis cannot establish temporal causation.
Gao et al. (2025)	Psychological adaptability	Digital-information-literacy self-efficacy	Work engagement	A statistically significant partial indirect effect was reported.	Unadjusted confounding and cross-sectional timing limit causal interpretation.
Kowitarttawatee and Limphaibool (2022)	Eastern mindfulness	Western mindfulness	Teacher resilience	A statistically significant partial indirect effect was reported.	Purposive sampling and unadjusted cross-sectional analysis limit causal interpretation.
Mehta (2021)	Autonomy, perceived ease of use, and perceived usefulness	Readiness to change/adopt online teaching	Perceived stress	Specific indirect effects through readiness were statistically significant.	Convenience/snowball sampling and cross-sectional timing preclude causal claims.
Padmanabhanunni et al. (2023)	Resilience	Role ambiguity (but not role conflict)	Life satisfaction, anxiety, and depression	Indirect effects through role ambiguity were reported; the role-conflict pathway was unsupported.	The proposed ordering was theory-driven rather than temporally observed.
Shi et al. (2025)	Psychological resilience	Digital competence	Online-teaching self-efficacy	A statistically significant partial indirect effect was reported.	Volunteer/snowball recruitment, omitted covariates, and cross-sectional timing limit causal interpretation.

### Strengths and limitations

Strengths include a fully re-audited final corpus, a DOI-level duplicate audit and corrected PRISMA arithmetic, full-text extraction for all 27 studies, explicit separation of construct routes, numerical reporting of theme coverage, design-specific MMAT appraisal without an overall score, and transparent correction of unsupported mechanism and moderator language. The study-level matrix, search audit log, and coding workbook permit readers to trace the selection chain and synthesis statements to their source records.

Several limitations remain. First, the common Boolean logic, databases, fields, dates, and yields were retained, but the complete platform-generated search histories were not; exact line-by-line replication of the original platform sessions is therefore not possible. Second, the search used two databases and may have missed studies indexed elsewhere or described without explicit resilience/adaptation language. Third, no review protocol was prospectively registered. Fourth, screening, synthesis, and MMAT decision-making were led by the primary author. A professor volunteered to assist with data extraction, processing, and supporting-evidence checks, but these contributions did not constitute independent duplicate screening, extraction, or appraisal; selection and interpretation bias therefore remain possible. Fifth, restricting full text to English may have reduced linguistic and geographical coverage. Sixth, theme frequencies do not measure effect size or certainty. Finally, heterogeneity and the predominance of cross-sectional evidence prevented meta-analysis and causal inference.

## Conclusion

Teacher resilience and adaptation in digital teaching are best represented in this evidence base as context-embedded patterns of psychological resources, digital capability, pedagogical agency, relational support, structural constraint, and wellbeing-related outcomes. The 27-study corpus contains seven indirect-effect models but no formal contextual moderation study, intervention trial, or prospective quantitative test of temporal relationships. Its principal contribution is a transparent evidence map that distinguishes what is frequently reported, what has been statistically modeled, and what remains untested. Stronger longitudinal, intervention, and context-sensitive research is needed before the field can explain how resilience and adaptation develop or determine which supports reliably improve them.

## Data Availability

The study-level extraction, theme coding, search audit log, corrected PRISMA counts and duplicate-pair audit, and complete Round-2 MMAT matrix are supplied with the revision as Supplementary File S1. The primary publications are available through the DOI or source links listed in the workbook and references.
